# Non-Incised Papilla Surgical Approach and Leukocyte Platelet-Rich Fibrin in Periodontal Reconstruction of Deep Intrabony Defects: A Case Series

**DOI:** 10.3390/ijerph18052465

**Published:** 2021-03-03

**Authors:** Guillermo Pardo-Zamora, José Antonio Moreno-Rodríguez, Antonio J. Ortiz-Ruíz

**Affiliations:** Department of General Dentistry and Implants, Faculty of Medicine and Dentistry, University of Murcia, 30008 Murcia, Spain; joseantonio171087@gmail.com (J.A.M.-R.); ajortiz@um.es (A.J.O.-R.)

**Keywords:** periodontitis, reconstructive surgery, regeneration, surgical flaps, non-incised papilla surgical approach, L-PRF

## Abstract

We present the preliminary results of the treatment of teeth with a deep, non-contained periodontal residual defect, vestibular bone dehiscence, and soft tissue recession, by combining an apical non-incised papilla surgical approach (NIPSA) to the defect and leukocyte platelet-rich fibrin (L-PRF) in the vestibular aspect. Four patients (upper left first premolar, upper left central incisor, upper right central incisor and upper right lateral incisor) have been treated. At one year of follow up, all cases showed a considerable reduction in the periodontal pocket depth, a gain in clinical attachment and no bleeding on probing, as well as an improvement in the marginal soft tissue minimizing soft tissue contraction (recession and/or loss of papilla) and improving soft tissue architecture. NIPSA plus L-PRF seem to improve clinical outcomes in deep non-contained intrabony defects associated with soft tissue recession.

## 1. Introduction

Evidence shows that teeth with deep periodontal pockets and advanced clinical attachment loss can successfully be treated by periodontal surgical reconstruction and maintained over time [1,2,3]. Although current minimally-invasive techniques for the treatment of intrabony defects are highly successful, both in eliminating the periodontal pocket and in the significant gain in clinical attachment, many studies have shown post-surgical contraction of the marginal soft tissue, with incomplete resolution of the intrabony component of the defect [4,5,6,7,8,9,10,11,12,13,14,15,16,17,18,19].

In most minimally-invasive periodontal surgical reconstruction techniques, access to the defect is made by incisions in the marginal aspect of the gingival tissue. The location of the incision in the marginal area presents some drawbacks, especially in some clinical situations where, due to the location of the incision at the base of the papilla and over the periodontal defect, to the anatomy of the interproximal area and the morphology of the defect, early healing may occur with dehiscence of the incision line. This increases the possibility of exposure of the area to be regenerated, with the risk of bacterial contamination and the formation of tissue composed of a rich inflammatory infiltrate, which compromises regeneration [4,9,10,13,17].

The non-incised papilla surgical approach (NIPSA) is a technique that accesses the periodontal defect from its apical aspect and was developed with the aim of achieving clinical improvements compared with marginal access techniques. NIPSA allows the structure of the interproximal tissue to be maintained without affecting or detaching the marginal tissues, which remain firmly inserted in the adjacent areas. In this way, the interproximal tissue acts as a “dome”, preserving the stability of the clot in the underlying defect and anticipating the collapse of marginal soft tissues [20,21].

In the case of non-contained deep periodontal defects associated with soft tissue recession, deep bone dehiscence, or poorly positioned teeth outside the bone contour, whether protruded or extruded, the technique is modified by adding a connective tissue graft (CTG) as a type of vestibular wall [22]. This allows the space to be maintained horizontal and the defect protected from horizontal collapse; CTG improves the mechanical stability and resistance to marginal forces [23], retards apical migration of the epithelium and, therefore, prevents the re-establishment of the periodontal pocket and improves soft tissue quality and quantity [24]. 

Platelet and leukocyte-rich fibrin (L-PRF) was introduced in 2001 as a therapeutic adjuvant to improve wound healing and tissue regeneration in oral surgery [25]. A recent systematic review found that L-PRF has a positive effect on soft tissue regeneration in various fields of medicine and dentistry [26]. The application of L-PRF membranes has also been associated with satisfactory results in the repair and regeneration of bone tissue [27,28]. In addition, clinical studies have demonstrated the regenerative capacity of L-PRF in regenerative periodontal surgery and mucogingival surgery [29]. 

The aim of this study was to present the preliminary results of a modification in the NIPSA technique, associating L-PRF, in the treatment of teeth with advanced loss of periodontal support, in non-contained periodontal defects associated with vestibular bone dehiscence.

## 2. Materials and Methods

We report four cases, all in females who were former smokers without systemic disease: the age range was 37 to 62 years. All had advanced periodontal disease treated non-surgically and complied with a periodontal maintenance phase of ≥12 months. All patients, after receiving a full description of the periodontal surgical procedure, signed informed consent in full accordance with the guidelines of the Helsinki World Medical Association Declaration and the revision of the 2013 Good Clinical Practice Guides. Each patient had a deep non-contained periodontal residual defect with soft tissue recession and deep bone dehiscence. The morphology of each defect was confirmed intra-surgically. An upper left first premolar, upper left central incisor, upper right central incisor and upper right lateral incisor were treated. An initial periodontal examination was performed before surgery and at 12 months of follow up, using a periodontal probe (PCP UNC 15, Hu-Friedy), including probing pocket depth (PPD), measured from the gingival margin to the bottom of the pocket, recession (REC) measured on the buccal aspect midline, from the cemento-enamel junction (CEJ) to the gingival margin, clinical attachment level (CAL) measured from CEJ to the bottom of the pocket, bleeding on probing (BoP) and tip of the papilla (TP) as the distance from the CEJ to the tip of the papilla. In case of CEJ alterations following crown restoration, adjacent tooth CEJ is taken as reference.

### 2.1. Pre-Surgical or Anti-Inflammatory Phase

Two weeks before surgery, the marginal aspect of the residual periodontal pocket associated with the intrabony defect was accessed by means of ultrasound microtips (PS tip, perio Slim, EMS) and microcurettes (Mini Langer, LM-Instruments Oy) to reduce the bacterial load, decrease inflammation, and improve the soft tissue conditions for further surgery. We avoided delving deeper to avoid injury to fibers that might still be inserted and to prevent increased soft tissue recession. The root surface exposed to the oral environment was also instrumentalized: 0.12% chlorhexidine rinses (Perio-Aid ^®^, Dentaid, Barcelona, Spain) were scheduled one week before surgery and two grams of amoxicillin (Amoxicillin 1 g Normon ^®^, Madrid, Spain) were administered one hour before surgery.

### 2.2. Surgical Phase

**Figure 1:**
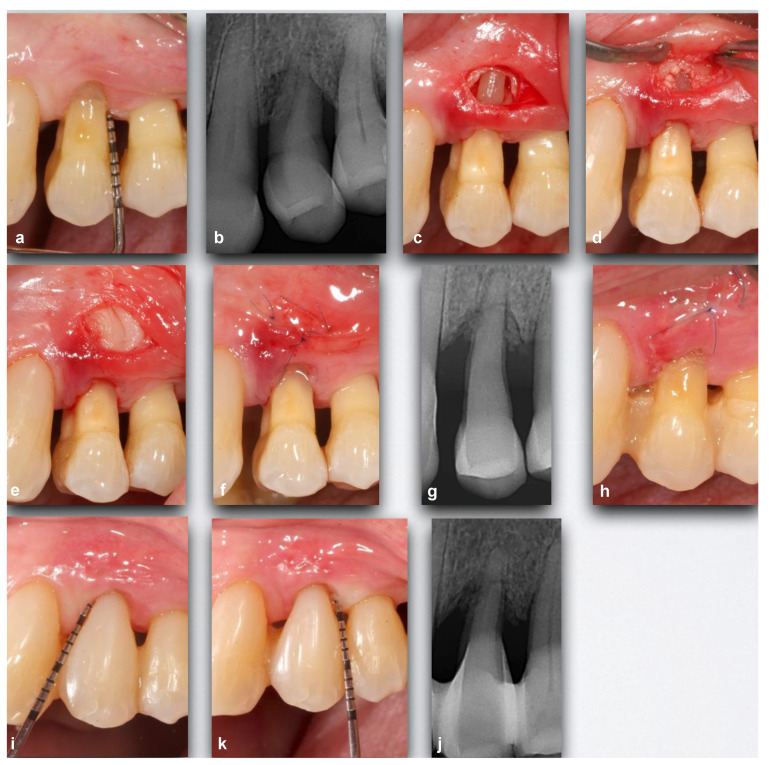
Case 24. (**a**,**b**) Upper left first premolar with advanced periodontal destruction and grade II mobility with two mesial and distal intra-bone defects. (**c**) Apical incision exposing intrabony defects and lack of vestibular cortical plates. (**d**,**e**) Biomaterials and adapted platelet and leukocyte-rich fibrin (L-PRF) membrane. (**f**,**g**) Suture and post-surgical X-ray. (**h**) Closure at the first attempt seven days post-surgery. (**i**–**k**) 12 months after surgery. A composite restoration was performed for aesthetic reasons. Significant reduction in probing pocket depth (PPD) and healthy soft tissues with acceptable scalloping. X-ray: resolution of intrabony defects.

**Figure 2:**
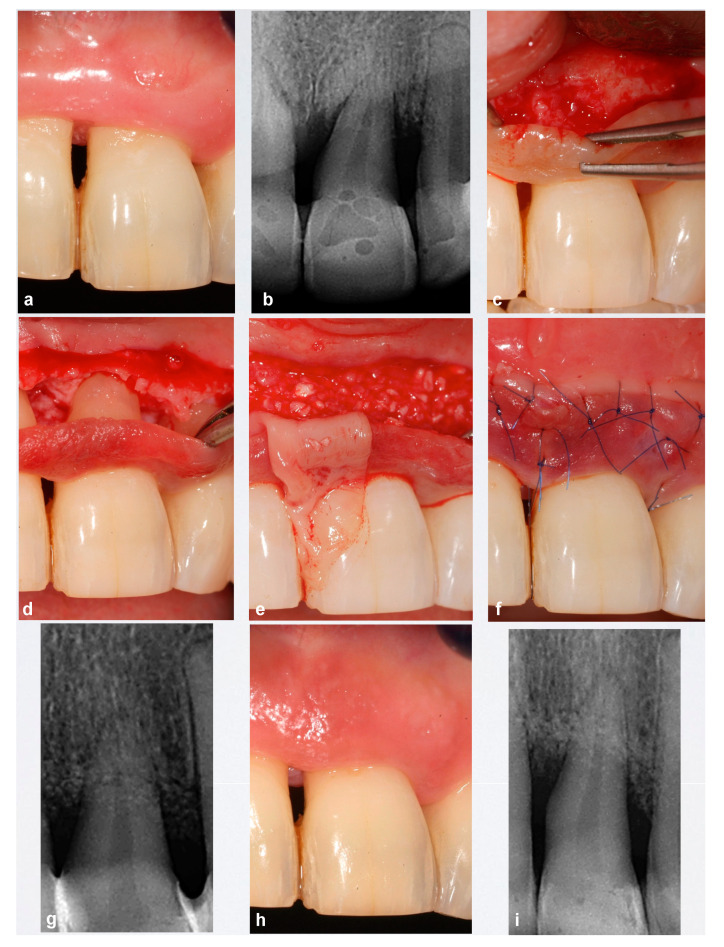
Case 21. (**a**,**b**) Upper left central incisor with advanced periodontal destruction and grade II mobility with two mesial and distal intra-bone defects. (**c**,**d**) Apical incision exposing intrabony defects and lack of vestibular cortical plate. (**e**) Biomaterials and adapted L-PRF membrane. (**f**,**g**) Suture and post-surgical X-ray. (**h**,**i**) Clinical view and X-ray 12 months after surgery.

Periodontal defects were accessed and tissues managed using NIPSA [20,21] through a single incision apical to the defect (Figure 1 and Figure 2). The incision, whether horizontal or oblique, was made in the mucosa on the cortical plates of the alveolar bone, apically to the bone crest delimiting the periodontal defect and away from the rooted tissue, without affecting the marginal tissues. The incision was extended mesiodistally as little as possible, but sufficient to allow access to and treat the defect. Subsequently the coronal tissue was raised to the incision at full thickness, maintaining the pre-surgical structural integrity of the marginal tissues, allowing access to the defect and maintaining a firm dome of fibrous tissue as a ceiling in the defect to be treated. 

The deep intermediate area of the periodontal pocket was treated because the marginal area was treated in the pre-surgical phase. The granulation tissue of the bone walls was disinserted using a micro curette or micro scalpel blade and that of the base of the papilla with micro scissors. The granulation tissue and periodontal pocket were removed and, before the application of biomaterials, traction was used to move the suprabony component of the defect (supra-alveolar soft tissue) towards the coronal. 

**Figure 3:**
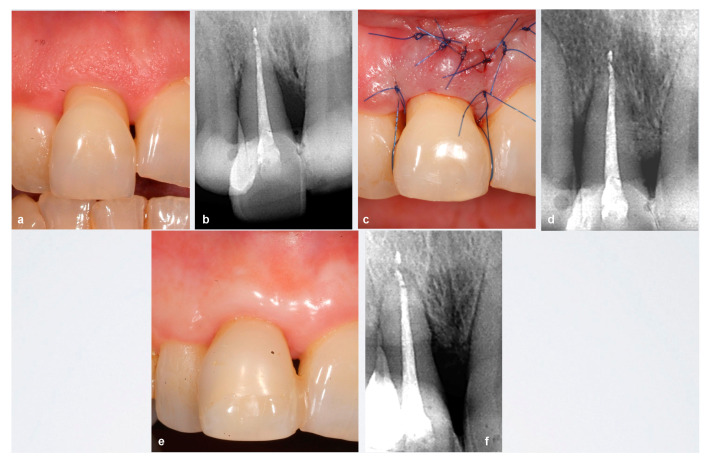
Case 11. (**a**,**b**) Upper right central incisor extruded with advanced periodontal destruction and grade II mobility with mesial intra-bone defect. (**c**,**d**) Suture and post-surgical X-ray. (**e**,**f**) 12 months after surgery. A composite restoration was performed for aesthetic reasons. X-ray: resolution of intrabony defect.

We applied 24% ethylene diamine tetra-acetic acid gel (PrefGel, Straumann^®^, Basel, Switzerland) to the root for two minutes. Subsequently, enamel matrix protein derivative (EMD) (EMD; Emdogain, Straumann^®^, Basel, Switzerland) was applied to the root, followed by a mixture of porcine collagenized bone xenograft (Gen-Oss, Osteobiol Tecnoss^®^ Dental s.r.l., Torino, Italy), EMD, and the liquid portion of L-PRF. We positioned a 1 mm thick L-PRF membrane on the vestibular aspect of the defect as a vestibular wall, which was fixed to the palatine firm tissue by internal mattress sutures, using 6/0 sutures of polyglycolic-caprolactone acid (Serafast^®^, Serag Wiessner, Naila, Germany). L-PRF membranes were prepared using the following protocol: 20 cc of blood are extracted from the ulnar vein of the right arm of the patient, which are centrifuged according to protocol at 2700 rpm for 12 min. Each test tube has a capacity of 10 cc and does not have any type of chemical added. Once the centrifugation process is finished, the fibrin clots are removed from the test tubes. These have the red series attached, so it is necessary to make a slight scraping with a spatula to separate them. Once the fibrin clots have been released, including platelets and leukocytes, they are placed on a perforated tray and a pressure sheet is placed on them for approximately 3 min to dislodge all the exudate from the clots that remains at the bottom of the tray, leaving them prepared as membranes. This exudate, which is rich in glycoproteins such as vitronectin, fibronectin, and thrombospondin, is ideal for hydrating the graft materials, providing growth factors with a natural vehicle at an appropriate pH [30,31]. Finally, the incision line was sutured according to the usual NIPSA protocol [20,21], except in one of the cases (Figure 3) in which, in addition to suturing the apical incision, double-crossed sutures were applied to stabilize the entire buccal soft- tissue complex.

**Figure 4:**
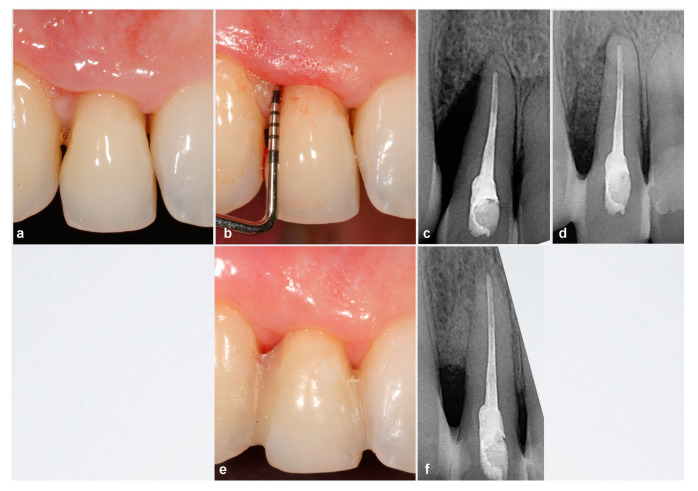
Case 12. (**a**–**c**) Clinical and radiographic examinations revealed an upper right lateral incisor with distal intra-bone defect reaching the apex of the tooth. The soft tissue showed a nonscalloped architecture as a result of chronic inflammation. (**d**) Post-surgical X-ray. (**e**,**f**) 12 months after surgery. Harmonious scalloped gingiva with physiologically healthy interdental papillae height resulting in form-controlled inflammation and coronal displacement of the papillae. X-ray: resolution of intrabony defect.

All the treated teeth had significantly increased mobility (grade II) so a temporary splinting was performed the same day of the regeneration surgery to prevent mobility during the healing phase of a regenerative periodontal procedure and may be removed later after healing. At 1 year, hypermobility was tested removing the resin splints. The resin splint was re-applied or not re-applied in agreement with the patients after a period of evaluation of the desplinted units for comfort and function [32,33,34]. In three of the four cases, splinting had to be maintained after 12 months (Figure 4). Furthermore, all contact in lateral excursions or protrusions was eliminated while maintaining only slight axial contact.

### 2.3. Post-Surgical Phase

Rinses were prescribed twice daily with 0.12% chlorhexidine digluconate (Perio-Aid^®^, Dentaid, Barcelona, Spain) for at least 4 weeks and ibuprofen 600 mg (Norvectan^®^, Laboratorio de Aplicaciones Farmacodinámicas, S.A. Barcelona, Spain) to control pain and inflammation. Post-surgical norms and hygiene techniques were explained. The sutures were removed within seven days of surgery. Follow-up checks were made at weeks 1, 2, 3 and 4 and months 3, 6 and 12.

## 3. Results

Complete closure was achieved at one week of healing in all four consecutive cases. All cases had positive pre-surgical BoP, which was negative at 12 months. Table 1 shows the clinical parameters at baseline and 12 months of follow-up. There was a mean PPD reduction of 4.75 ± 1.08 mm and a mean CAL gain of 5.5 ± 0.5 mm. The mean reduction in REC was 0.75 ± 1.08 mm and TP showed a mean coronal displacement of 0.25 ± 0.4 mm. 

## 4. Discussion

We present four cases with an advanced loss of periodontal support, non-contained periodontal defects associated with vestibular bone dehiscence, which were treated using NIPSA and L-PRF. 

In these cases, former smokers were selected. In periodontal reconstruction surgery wound healing results were significantly worse in smokers as reported in different studies [35,36]. Furthermore, deep periodontal lesions (PPD > 6 mm and CAL > 9 mm) were selected. Deep lesions are frequently associated with non-contain defects and buccal bone dehiscences or supra-alveolar type-defect. This type of defect means an ideal situation to gain access by NIPSA from the buccal aspect maintaining the papilla and marginal tissue arquitecture intact in the vertical aspect. In all four cases, a reduction in PPD, a gain in CAL and the filling of the defect was achieved, in addition to the preservation of marginal soft tissues at 12 months of follow up, although in this type of defect it is difficult to maintain space for clot stabilization and maintain stability in the area during healing. NIPSA achieved the necessary stability due to the structural integrity of the interproximal soft tissue on the periodontal defect, which provided a ceiling or dome of fibrous tissue [20,21].

Marginal approaches, by influencing and disinserting the papilla and marginal soft tissue sulcularly, facilitate marginal and vertical collapse. Micromovements and mechanical forces generated during chewing and brushing may cause problems in clot-root adhesion and compromise periodontal regeneration. In addition, the difficulty in stabilizing marginal tissues against the root surface creates aesthetic problems associated with soft tissue retraction after healing [23].

In the case of deep non-contained periodontal defects associated with soft tissue recession, deep bone dehiscence, or poorly positioned teeth (protruded or extruded outside the bone contour), NIPSA was modified by adding a connective tissue graft (CTG) as a vestibular wall [22]. In this way, the space can be maintained horizontally, and the defect protected from horizontal collapse [24]. In the four cases we present, the defect was filled with porcine collagenized xenograft, EMD, and L-PRF and we used a 1 mm thick L-PRF membrane as a type of vestibular wall, as an alternative to CTG. The use of the L-PRF membrane prevented post-surgical recession [37], increased wound stability by reducing the micro-movement of the flap to the clot-dentin complex [38,39] and delayed epithelial migration to the defect [40]. The application of EMD is justified by its capacity to induce and accelerate differentiation of cells of periodontal origin [41]. The additional use of a bone graft seems to improve and enhance its properties [42,43]. 

L-PRF may be considered as a live tissue graft due to its cellular content and its constant release of growth factors for more than 7 days [44]. In vitro studies have demonstrated its ability to stimulate the proliferation and migration of periodontal fibroblasts [45]. Likewise, L-PRF has been shown to have a positive effect when applied in periodontal surgery for the treatment of intrabony defects [46,47] and the regeneration of grade II furcation defects [48]. A recent meta-analysis found a significant 1.1 mm PPD reduction, a 1.2 mm CAL gain, and a 1.7 mm bone filling in intrabony defects in favor of open flap debridement + L-PRF as a periodontal defect filler, and the use of L-PRF membranes to cover the defects in a similar way to a membrane in guided tissue regeneration compared with open flap debridement alone [29]. Applying an L-PRF membrane can improve wound stability and protect the blood clot and graft complex by controlling flap micromovements during healing [38]. 

The use of L-PRF in regenerative and mucogingival therapy can improve the quality and quantity of soft tissues [29]. In mucogingival plastic surgery, there are differing results in recession coverage when comparing coronal displaced flaps with coronal displaced flaps + L-PRF. Although most studies found no significant differences, the L-PRF combination was superior in all parameters recorded [49,50,51]. A recent systematic review found a mean root coverage of 86.5% at 6 months after treatment with coronal displaced flaps + L-PRF [29]. The cases treated in our study showed no alterations in the integrity of marginal soft tissues or papillae either immediately after surgery or during the healing period, and the recession depth was reduced.

In these cases, with advanced periodontal destruction and deep bone peak location, the NIPSA approach facilitates soft tissue preservation. By this approach, the maintenance of the marginal soft tissue integrity may favor the resistance to marginal forces during the healing period and preserve healing area integrity and space stability in the vertical aspect. However, this approach has a limitation in the treatment with a high lingual extension where the decontamination may be done without a direct vision.

LPRF was used instead of CTG, avoiding a second surgical area and decreasing the procedure morbidity. However, L-PRF has poorer physical properties than CTG and it may be associated with a graft filling material to give support in the horizontal aspect.

## 5. Conclusions

The preliminary results after the use of NIPSA plus L-PRF showed considerable improvements in clinical parameters, with complete elimination of the periodontal pocket and a gain in periodontal attachment. There was also an improvement in the soft tissues, with reductions in the recession and papilla loss that normally occur after the treatment of deep periodontal defects.

## Figures and Tables

**Table 1:** Clinical parameters.

	Tooth	PPD		CAL		REC		TP	
		Baseline	12 m	Baseline	12 m	Baseline	12 m	Baseline	12 m
Case 1	24	7	4	12	7	5	3	−5	−5
Case 2	21	9	4	11	5	2	1	0	1
Case 3	11	8	3	10	4	2	1	2	2
Case 4	12	9	3	10	5	1	2	1	1
Mean ± SD		8.25 ± 0.95	3.5 ± 0.57	10.75 ± 0.95	5.25 ± 1.25	2.5 ± 1.7	1.75 ± 0.95	−0.5 ± 2.69	−0.25 ± 2.7

PPD—probing pocket depth; CAL—clinical attachment level; REC—recession; TP—tip of the papilla; SD—standard deviation.
